# Effect of High-Intensity Rosuvastatin vs. Combination of Low-Intensity Rosuvastatin and Ezetimibe on HbA1c Levels in Patients without Diabetes: A Randomized IDEAL Trial

**DOI:** 10.3390/jcm12186099

**Published:** 2023-09-21

**Authors:** Jeongcheon Choe, Sun-Hack Lee, Jinhee Ahn, Hyewon Lee, Jun-Hyok Oh, Junghyun Choi, Hancheol Lee, Kwangsoo Cha, Jinsup Park

**Affiliations:** Department of Cardiology and Medical Research Institute, Pusan National University Hospital, Busan 49241, Republic of Korea; purefountain@naver.com (J.C.); ycvred@gmail.com (S.-H.L.); reinee81@naver.com (J.A.); lhw1400@hanmail.net (H.L.); jhoh724@hanmail.net (J.-H.O.); mariahyeon@gmail.com (J.C.); glaraone@hanmail.net (H.L.); chakws1@hanmail.net (K.C.)

**Keywords:** rosuvastatin, ezetimibe, diabetes, HbA1c, combination, low-intensity

## Abstract

There is a dearth of studies investigating whether the combination of low-intensity statins with ezetimibe can reduce the risk of diabetes in patients requiring statin therapy. Therefore, we aimed to evaluate the effects of combination therapy on the prevention of glycated hemoglobin (HbA1c) elevation in patients without diabetes. Sixty-eight patients were randomly assigned in a 1:1 ratio to receive a combination of low-intensity rosuvastatin (5 mg/day) and ezetimibe (10 mg/day) or high-intensity rosuvastatin (20 mg/day). The primary endpoint was the absolute difference in the HbA1c levels at 12 weeks. The HbA1c level showed an overall elevation of 0.11% at 12 weeks compared to that at baseline (mean ± standard deviation: 5.78 ± 0.3%, 95% confidence interval [CI]: 5.86–6.07, *p* = 0.044). The HbA1c levels did not differ between the groups at 12 weeks (least square mean difference: 0.001, 95% CI: 0.164–0.16, *p* = 0.999). Our study found that the combination of low-intensity rosuvastatin and ezetimibe did not yield significant differences in HbA1c levels compared to high-intensity rosuvastatin alone after 12 weeks in patients without diabetes. This suggests that the combination of low-intensity rosuvastatin and ezetimibe may not be an effective strategy for preventing HbA1c elevation in patients without diabetes requiring statins.

## 1. Introduction

Major advances in the understanding and management of atherosclerosis over the past few decades have led to substantial reductions in morbidity and mortality arising from atherosclerotic cardiovascular disease (ASCVD). These advances have been largely driven by more effective risk factor management and the introduction of statin therapy, which significantly lowers low-density lipoprotein cholesterol (LDL-C) levels [1].

Diabetes significantly increases the risk of ASCVD, for which statin therapy has been recommended for primary and secondary prevention [2]. However, two meta-analyses have raised concerns about the potential links between statin therapy and the risk of diabetes, which have led to some modifications in the relevant guidelines and expert opinions for the management of dyslipidemias [3,4]. Notably, the JUPITER trial reported a modest increase in the incidence of diabetes in patients treated with rosuvastatin compared to those treated with a placebo despite a significant reduction in cardiovascular risk [5]. Statin therapy of high intensity and longer duration seems to be associated with an elevated risk of diabetes [6,7]. Moreover, patients with established risk factors, such as obesity or metabolic syndrome, are more likely to develop diabetes during statin therapy [8].

Ezetimibe, a cholesterol absorption inhibitor, acts synergistically with statins to reduce LDL-C levels by 23–24% [9]. The combination of ezetimibe and low-intensity statins can effectively manage LDL-C, reduce the required dose of statin, and mitigate the side effects associated with high-intensity statin therapy [10,11]. The efficacy data from a meta-analysis of randomized controlled trials (RCTs) suggest that combination therapy with a moderate-intensity statin and ezetimibe produces a greater reduction in LDL-C levels than that with high-intensity statin monotherapy [12]. A recent RCT validated the results of a meta-analysis, demonstrating that combination therapy with a moderate-intensity statin and ezetimibe can achieve greater reduction in LDL-C levels and, as secondary prevention, achieve LDL-C levels below 70 mg/dL in a higher proportion of patients [13]. Although numerous studies have investigated the efficacy and safety of combining moderate-intensity statins with ezetimibe for lowering LDL-C levels, no RCT has evaluated whether this combination can reduce the risk of diabetes in patients without diabetes requiring statin therapy.

Therefore, to address this knowledge gap, we conducted an RCT to evaluate the effect of combination therapy with low-intensity rosuvastatin at a dose of 5 mg and ezetimibe 10 mg in preventing the elevation of glycated hemoglobin (HbA1c) levels in patients without diabetes compared with high-intensity rosuvastatin monotherapy at a dose of 20 mg.

## 2. Materials and Methods

### 2.1. Study Design and Ethical Concerns

The impact of high-intensity rosuvastatin and a combination of low-intensity rosuvastatin and ezetimibe on HbA1c levels in patients without diabetes (IDEAL; KCT0007278) study was a single-center, prospective, open-label, randomized trial. This study compared the effects of the combination of low-intensity rosuvastatin and ezetimibe with those of high-intensity rosuvastatin monotherapy on glucose metabolism, especially the alterations in the HbA1c and lipid profiles, over 12 weeks in patients without diabetes who required statin therapy. The study design was approved by the Institutional Review Board of Pusan National University Hospital, Busan, Republic of Korea, and conducted in accordance with the principles of the Declaration of Helsinki. Informed consent was obtained from all participants. This trial was registered with the Clinical Research Information Service (CRIS) of the National Institutes of Health, Republic of Korea (identifier: KCT0007278).

### 2.2. Patients

Patients aged >18 years who required statin therapy at the time of enrollment were considered eligible for the study. The indications for statin therapy were as follows: (1) definitive diagnosis of ASCVD, such as coronary artery disease or cerebrovascular accident; (2) persistent elevation of LDL-C levels to >190 mg/dL despite intensive lifestyle modifications; (3) persistent elevation of LDL-C levels to >160 mg/dL with at least one cardiovascular risk factor; (4) persistent elevation of LDL-C levels to >130 mg/dL with evidence of atherosclerosis; (5) high-sensitivity C-reactive protein (hs-CRP) level > 2 ng/dL; and (6) coronary calcium score > 100 on coronary computed tomography (CT). Patients with serum HbA1c levels > 6.5% during the enrollment period and those who were treated with any type of glucose-lowering medication were excluded from the study. The other exclusion criteria included severe liver disease, myopathy, creatinine clearance <30 mL/min, galactose intolerance, and concomitant use of drugs that can influence serum lipid and glucose levels.

### 2.3. Randomization and Data Collection

Patients who required statin therapy were randomly assigned in a 1:1 ratio to receive either high-intensity rosuvastatin 20 mg/day (rosu20 group) or a combination of low-intensity rosuvastatin 5 mg/day and ezetimibe 10 mg/day (rosu5ezet10 group). Randomization was performed using computer-generated block randomization. The participants received education and advice on lifestyle modifications aimed at managing their lipid profiles. Other medications were administered as indicated, except for those that could affect the lipid profile and glucose metabolism. Clinical follow-ups were conducted at 6 and 12 weeks after enrollment. Information on the use of concomitant drugs and the clinical status of adverse drug reactions was obtained at each follow-up visit.

Laboratory assessments were conducted at baseline and at 6 and 12 weeks of follow-up. The HbA1c level was measured using a high-performance liquid chromatography assay; the glucose and lipid profiles were determined using the enzyme-linked immunosorbent assay; the hs-CRP level was assessed using latex agglutination; and plasma insulin levels were measured using an immunoradiometric assay. The quantitative insulin sensitivity check index (QUICKI), which is generally used as a surrogate marker of insulin sensitivity, was calculated based on the serum insulin and fasting glucose levels using the following equation: QUICKI = 1/(log [insulin, U/mL] + log [glucose, mg/dL]).

### 2.4. Endpoints

The primary endpoint was the difference in the HbA1c levels between the combination therapy and monotherapy groups at 6 and 12 weeks. The secondary endpoints included differences in plasma insulin and fasting glucose levels, QUICKI values, and changes in the hs-CRP levels and lipid profile, including LDL-C, high-density lipoprotein cholesterol (HDL-C), and triglyceride (TG) levels.

### 2.5. Statistical Analysis

Based on the results of a previous study [14], we estimated that a sample size of 33 patients per group would be required to detect a statistically significant absolute difference of 0.15% in HbA1c level with 80% power at a two-sided significance level of 0.05. To account for potential dropouts, we assumed a dropout rate of 5% and enrolled 70 patients.

Continuous variables were analyzed using Student’s *t*-test or Wilcoxon rank-sum test, as appropriate. Categorical variables were analyzed using the chi-squared test or Fisher’s exact test. We hypothesized that the absolute difference in the HbA1c levels from baseline to 12 weeks would be lower in the group treated with the combination of low-intensity rosuvastatin and ezetimibe than that in the high-intensity rosuvastatin group. Multiple linear mixed-effects models were constructed to analyze and compare the absolute changes in HbA1c levels and other dependent variables between the two groups from baseline to 6 and 12 weeks. These models incorporated both the fixed and random effects of absolute changes in HbA1c levels and other dependent variables. The models estimated the parameters using the restricted maximum likelihood method, which is employed for non-normally distributed data. The least square (LS) means, 95% confidence intervals (CIs), and *p*-values were calculated from multiple linear mixed-effects models that accounted for the effects of time, the interaction between time and treatment, and the interaction between time and baseline risk factors (hypertension, current smoker, body mass index (BMI), and atherosclerosis) and evaluated using the Benjamini–Yekutieli (BY) method. The BY method and multivariate t-distribution were used to control the false discovery rate (FDR) for multiple comparisons. The BY method is a step-down procedure that adjusts *p*-values to control the FDR at a pre-specified level. Multivariate t-distribution is a probability distribution that can be used to calculate the *p*-values for multiple comparisons. Subgroup analyses were conducted for pre-specified groups of prediabetes (HbA1c ≥ 5.8%) vs. normoglycemia (HbA1c < 5.8%), women vs. men, age ≥75 years vs. age <75 years, and high BMI (≥25 kg/m^2^) vs. normal BMI (<25 kg/m^2^). Analyses were conducted using R version 3.1.1 with the nlme and emmeans libraries. 

## 3. Results

**Figure 1:**
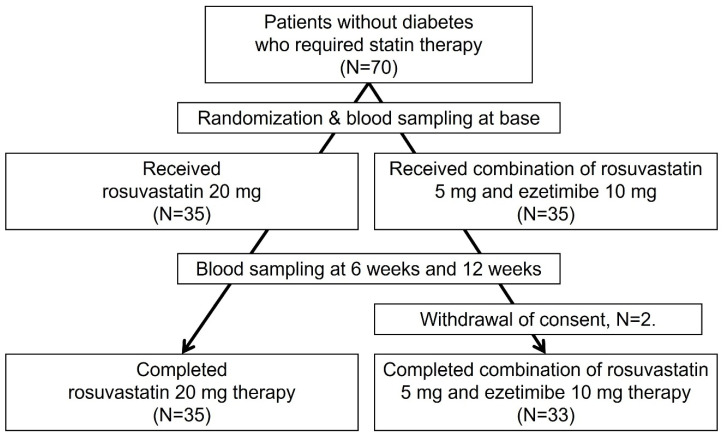
Study flow chart.

Seventy patients were enrolled between February 2019 and June 2022. Of these, two patients withdrew consent and were excluded from the final analysis. The remaining 68 patients were randomized to receive either high-intensity rosuvastatin 20 mg/day (rosu20 group, n = 35) or a combination of low-intensity rosuvastatin 5 mg/day and ezetimibe 10 mg/day (rosu5ezet10 group, n = 33). No clinically significant adverse events were reported, although mild LFT elevation (less than three times the normal value) was noted in 25% of patients. There was no significant LFT elevation and no changes in the study drugs in either treatment group during the study period. No instances of clinically relevant muscle pain were observed; however, approximately 30% of patients experienced some kind of muscle pain, which was not associated with elevated creatine kinase (CK) levels (Figure 1).

The baseline clinical characteristics, laboratory findings, and medications were similar between the treatment groups (Table 1). The mean age and BMI of the participants were 62 years and 25.6 kg/m^2^, respectively, and 76% of the participants were men. Half of the patients received antihypertensive medications, and 25% were current smokers. The most common indication for statin therapy was a high coronary artery calcium score on CT.

### 3.1. Absolute Changes in Glucose Metabolism and Lipid Profiles

**Figure 2:**
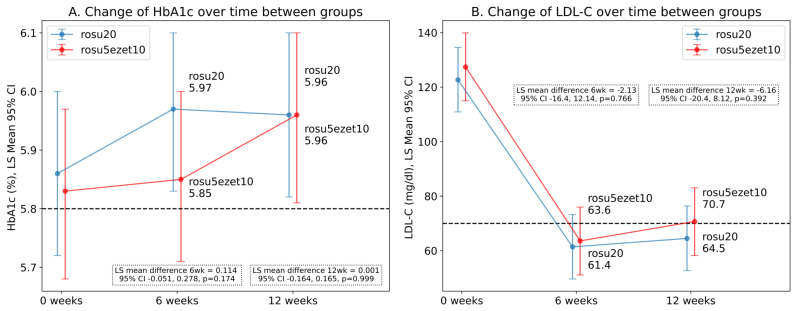
The least square means and the differences in HbA1c (**A**) and LDL-C (**B**) levels from baseline to 6 and 12 weeks in patients who received rosuvastatin 20 mg (rosu20) or a combination of rosuvastatin 5 mg and ezetimibe 10 mg (rosu5ezet10). Dotted lines indicate the cut-off value for prediabetes (**A**) and optimal therapeutic range (**B**). The LS mean, SE, 95% CI, and *p*-values were calculated from the multiple linear mixed-effects models that accounted for the effects of time, interaction between time and treatment, and interaction between time and baseline risk factors. LS, least square; LDL-C, low-density lipoprotein cholesterol; HbA1c, hemoglobin A1c; SE, standard error; CI, confidence interval.

Overall, the HbA1c level showed an increase of 0.11% at 12 weeks compared to that at baseline (mean ± SD: 5.78 ± 0.3%, 95% CI: 5.86–6.07, *p* = 0.044). The LS mean HbA1c level increased insignificantly from 5.8 ± 0.3 to 5.96% in the rosu20 group (*p* = 0.157) and from 5.83 to 5.96% in the rosu5ezet10 group, implying a weak relationship (*p* = 0.065). The HbA1c levels at 6 and 12 weeks did not differ significantly between the two groups (Figure 2a). The fasting glucose levels decreased significantly from 134 mg/dL to 106 mg/dL (LS mean: −28.7 mg/dL, *p* < 0.001), while the insulin levels (increased from 10 µU/mL to 12.2 µU/mL, LS mean: 2.15 µU/mL, *p* = 0.221) did not. The QUICKI values increased from 1.40 at baseline to 1.52 at 12 weeks (LS mean: 0.11, *p* = 0.02) only in the rosu20 group (LS mean: 0.15, *p* = 0.01). There was no significant difference in glucose metabolism between the groups at baseline or at 6 and 12 weeks (Table 2).

Both treatment strategies resulted in a significant reduction in LDL-C levels throughout the study period. The overall reduction in the LDL-C level was 57 mg/dL at 12 weeks (from 125 mg/dL to 68 mg/dL, *p* < 0.001). The rosu20 group exhibited a mean LDL-C reduction of 58 mg/dL (from 123 mg/dL to 65 mg/dL, *p* < 0.001), whereas the rosu5ezet10 group exhibited a mean LDL-C reduction of 57 mg/dL (from 127 mg/dL to 71 mg/dL, *p* < 0.001), albeit without significant inter-group differences at 6 and 12 weeks (Figure 2b). Concurrently, a prominent increase was observed in the HDL-C levels (from 46 mg/dL to 51 mg/Dl, LS mean: 5 mg/dL, *p* < 0.001), and the effect on the elevation of HDL-C levels was 1.7 times higher in the rosu20 group than that in the rosu5ezet10 group (LS mean: 6.68 mg/dL, *p* < 0.001 vs. LS mean: 3.98 mg/dL, *p* = 0.016). There was no decrease in the TG (LS mean: −15 mg/dL, *p* = 0.28) and hs-CRP (LS mean: −0.385 mg/dL, *p* = 0.267) levels during the study period. These results suggest that both treatment regimens were effective in improving the lipid profile. However, despite these substantial alterations, no significant differences were detected in the lipid profiles between the treatment groups at baseline and at 6 and 12 weeks (Table 3).

### 3.2. Percent Changes in Glucose Metabolism and Lipid Profiles

**Figure 3:**
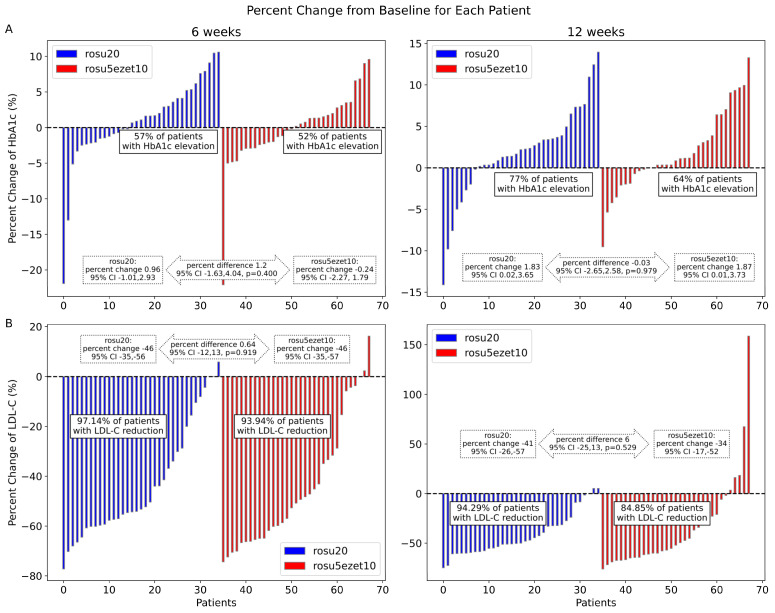
The percent change in HbA1c (**A**) and LDL-C (**B**) levels from baseline to 6 and 12 weeks in each patient who received rosuvastatin 20 mg (rosu20) or a combination of rosuvastatin 5 mg and ezetimibe 10 mg (rosu5ezet10).

The LS mean percent change in the HbA1c levels increased significantly over time from 0.37% at 6 weeks to 1.84% at 12 weeks (LS mean percent change: 1.85%, 95% CI: 0.23–3.46%, *p* = 0.021). The percent change in HbA1c levels increased continuously in the rosu20 group (0.96% at 6 weeks and 1.83% at 12 weeks), whereas it decreased slightly in the rosu5ezet10 group at 6 weeks (−0.24%), followed by an elevation at 12 weeks (1.87%). At 6 weeks, 57% of patients in the rosu20 group and 52% of patients in the rosu5ezet10 group had increased HbA1c levels, while at 12 weeks, 77% of patients in the rosu20 group and 64% of patients in the rosu5ezet10 group showed increased HbA1c levels. There were no differences in the percent change in HbA1c levels between the groups at 6 and 12 weeks (Figure 3a).

An LS mean percent reduction in LDL-C level was observed from baseline to 12 weeks (44% at 6 weeks and 38% at 12 weeks, mean percent difference: 38%, 95% CI: 29–47 %, *p* < 0.001). Both treatment groups showed similar reductions in LDL-C levels during the study period. At 6 weeks, 2.86% and 6.06% of patients in the rosu20 and rosu5ezet10 groups, respectively, did not experience a reduction in LDL-C levels. At 12 weeks, the gap between the groups widened, with 5.71% of patients in the rosu20 group and 15.15% of patients in the rosu5ezet10 group not experiencing a reduction in LDL-C levels (Figure 3b).

The LS mean, SE, 95% CI, and *p*-values were calculated from multiple linear mixed-effects models that accounted for the effects of time, interaction between time and treatment, and interaction between time and baseline risk factors.

LDL-C, low-density lipoprotein cholesterol; HbA1c, hemoglobin A1c; SE, standard error; CI, confidence interval.

### 3.3. Analyses in Pre-Defined Subgroups

**Figure 4:**
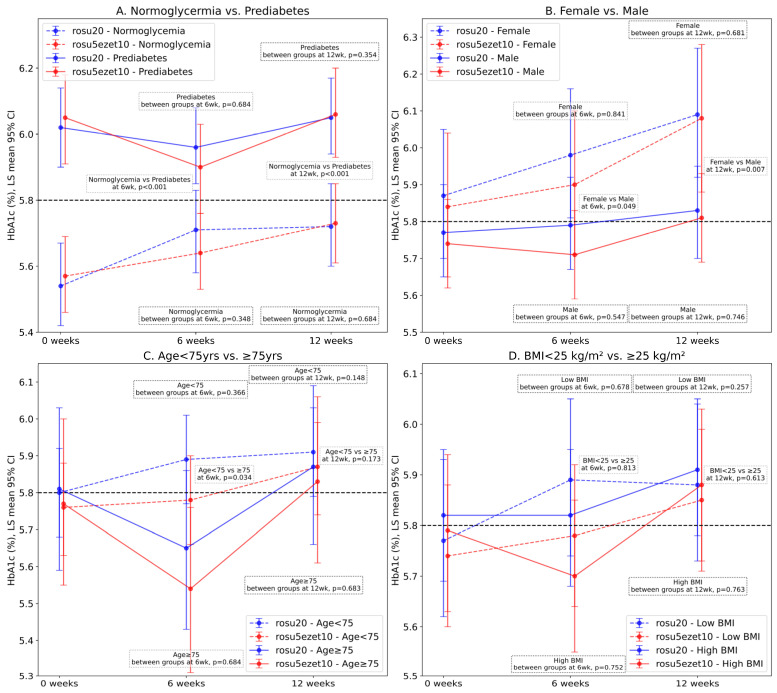
Least square means and their differences in HbA1c levels from baseline to 6 and 12 weeks in pre-defined subgroups stratified according to prediabetes (**A**), sex (**B**), age (**C**), and BMI (**D**) in patients who received rosuvastatin 20 mg (rosu20) or a combination of rosuvastatin 5 mg and ezetimibe 10 mg (rosu5ezet10). The LS mean, SE, 95% CI, and *p*-values were calculated from multiple linear mixed-effects models that accounted for the effects of time, interaction between time and treatment, and interaction between time and baseline risk factors. Definitions: prediabetes, baseline HbA1c level > 5.8%; obesity, baseline BMI > 25 kg/m^2^. LS, least square; BMI, body mass index; HbA1c, hemoglobin A1c; SE, standard error; CI, confidence interval.

The increase in the HbA1c level from baseline to 12 weeks was significantly higher in the normal subgroup (LS mean: 0.18%, *p* = 0.03 and LS mean: 0.16%, *p* = 0.042 for the rosu20 and rosu5ezet10 groups, respectively) than that in the prediabetes subgroup (LS mean: 0.036%, *p* = 0.853 and LS mean: 0.014%, *p* = 0.98 for the rosu20 and rosu5ezet10 groups, respectively). The HbA1c level was significantly higher in female patients at 12 weeks than in male patients (LS mean: 0.22%, *p* = 0.038 and LS mean: 0.24%, *p* = 0.042 for the rosu20 and rosu5ezet10 groups, respectively). Overall, patients aged <75 years exhibited a significant elevation in the HbA1c level from baseline to 12 weeks (LS mean: 0.11%, *p* = 0.033) compared to those aged >75 years (LS mean: 0.06%, *p* = 0.81). No increase in HbA1c levels was observed in patients with BMI > 25 kg/m^2^ (LS mean: 0.09%, *p* = 0.291 and LS mean: 0.09%, *p* = 0.419 for the rosu20 and rosu5ezet10 groups, respectively) during the study period. There were no significant differences in the HbA1c level between the subgroups stratified according to prediabetes/hyperglycemia, age, BMI, and sex at 6 and 12 weeks (Figure 4).

## 4. Discussion

Despite an overall increase in the HbA1c levels and a decline in LDL-C levels throughout the study period, there were no significant differences in HbA1c or LDL-C levels between the treatment groups at 12 weeks of statin therapy. High-intensity rosuvastatin monotherapy bore a stronger association with increased insulin sensitivity and HDL-C levels than the combination of low-intensity rosuvastatin and ezetimibe. In patients with normoglycemia at baseline, female sex and age <75 years were associated with increased HbA1c levels during statin therapy. However, BMI was not associated with increased HbA1c levels, irrespective of the treatment strategy.

There is concrete evidence that statin therapy is associated with a higher risk of developing diabetes compared to non-statin therapy [7]. However, the association between the statin dose and the risk of diabetes remains inconclusive, as evidenced by our study, which found similar HbA1c levels between patients who received high- and low-intensity statin therapies. Ko et al. [15] compared the efficacy of high-intensity and moderate-intensity statin therapy and found that the risk of diabetes was not associated with the statin dose in patients with myocardial infarction. Some evidence suggests that ezetimibe may increase the risk of developing diabetes. The relationship between lipid-induced oxidative stress and insulin resistance could explain the adverse effects of ezetimibe on glucose metabolism [16]. This finding is concordant with that of another study in which the ezetimibe group exhibited increased urinary 8-isoprostane levels, which were negatively correlated with insulin sensitivity metrics, such as the Matsuda index and QUICKI. Additionally, the increase in the expression of immune-related genes in the liver of ezetimibe-treated patients is indicative of oxidative stress induced by ezetimibe [17]. Lotta et al. [18] conducted a Mendelian randomization study and found an association between ezetimibe exposure and elevated risk of type 2 diabetes, which was observed in individuals with genetic variants located near *NPC1L1*. These findings are consistent with the results of an RCT that showed an increase in HbA1c levels associated with ezetimibe treatment [17]. However, a meta-analysis [19] failed to elucidate a clear association between a combined ezetimibe and statin regimen and the risk of diabetes. A post-hoc analysis from the IMPROVE-IT trial, a large RCT that compared the effects of ezetimibe plus simvastatin versus simvastatin alone in patients with dyslipidemia, also failed to find a higher risk of developing type 2 diabetes with the former regimen [20]. Therefore, current evidence on the association between ezetimibe and diabetes risk remains unclear, even though ezetimibe, a cholesterol absorption inhibitor, lowers LDL-C levels without affecting blood sugar levels. Further studies are required to determine the existence of a causal relationship between these two factors. Patients taking ezetimibe should be aware of the potential risk of developing diabetes, and their blood glucose levels should be monitored closely.

In our study, the mean reduction in LDL-C levels was similar for both treatment strategies, i.e., high-intensity rosuvastatin vs. a combination of low-intensity rosuvastatin and ezetimibe. However, there was a high variance in the LDL-C levels at 12 weeks with the combination of rosuvastatin and ezetimibe, which could lead to a reduction in the LDL-C-lowering effect of statin therapy. A meta-analysis of RCTs revealed a similar reduction in LDL-C levels with high-intensity statin therapy and the combination of low-intensity statins and ezetimibe; however, the combination group tended to have a lower degree of reduction in LDL-C levels (−4.1 mg/dL) [21]. Westerink et al. [22] assessed the difference in endothelial function between high-intensity simvastatin and the combination of low-intensity simvastatin and ezetimibe and found no differences in the LDL-C reduction achieved by the two regimens, although the combination group tended to show a lower degree of reduction (1.81 mmol/L for high-intensity statin vs. 1.79 mmol/L for combination therapy). Furthermore, Piorkowski et al. [23] discovered a reduction in the pleiotropic effect of statins in patients with coronary artery disease who received a combination of low-intensity statins and ezetimibe. Kim et al. [13] conducted an RCT, which revealed that the combination of moderate-intensity statin with ezetimibe had comparable long-term clinical outcomes and that combination therapy was associated with a higher proportion of patients with LDL-C levels below the recommended target and a higher compliance rate. These findings suggest that a higher dose of statins in combination with ezetimibe may be more beneficial for lowering LDL-C levels than a combination of low-dose statins with ezetimibe in routine clinical practice.

As shown in our study, the HDL-C levels increased in a dose-dependent manner with rosuvastatin therapy [24]. In terms of the effect of statins on insulin sensitivity, although studies have hypothesized that statins could exert a deleterious impact on beta cell function [25], there is mixed evidence about the effect of statins on insulin sensitivity in patients without diabetes; for instance, rosuvastatin is thought to have a neutral effect on new-onset diabetes [26]. Several studies have shown that younger patients and patients with factors predisposing toward diabetes development, such as metabolic syndrome, high BMI, and female sex, face an increased risk of developing diabetes while receiving statin therapy [27,28,29,30]. Our study showed significant elevation in HbA1c levels in patients with normoglycemia compared to patients with prediabetes from baseline to 12 weeks, possibly because patients diagnosed with prediabetes may have participated in more intensive lifestyle modifications. Although the principal mechanisms underlying the risk of diabetes with statin treatment have not been elucidated, Yang and Schooling’s [27] Mendelian randomization study revealed that half of the risk of diabetes associated with statin therapy could be attributed to statin-induced weight gain; however, a meta-analysis of RCTs revealed no difference in weight gain between high-intensity and moderate-intensity statin treatment [6]. This discrepancy could explain the findings of our study, which showed no increase in weight and similar elevation in HbA1c levels between high-intensity statin monotherapy and the combination of low-intensity statin and ezetimibe. 

Our study found that the combination of low-intensity rosuvastatin and ezetimibe was not effective in preventing HbA1c level elevation. Moreover, concerns exist that this combined regimen may not be sufficient to lower lipid levels. Furthermore, our study had several limitations. First, we primarily focused on identifying differences in HbA1c levels between the two treatment strategies. Therefore, the sample size may have been insufficient for meaningful comparisons of other blood parameters, such as glucose metabolism markers and lipid profile, as well as a subgroup analysis. Second, this study was conducted at a single institution, which may limit the generalizability of our findings to other settings. Third, the follow-up period was short, which may have limited the detection of long-term differences in HbA1c levels and the actual incidence of diabetes between the treatment strategies. Future multicenter studies with larger sample sizes and longer follow-up periods are needed to confirm our findings and further investigate the potential benefits and risks of different statin treatment strategies in patients without diabetes.

## 5. Conclusions

Our study found no significant differences in the HbA1c levels after 12 weeks of treatment with rosuvastatin 20 mg or a combination of rosuvastatin 5 mg and ezetimibe 10 mg in patients without diabetes. Our findings suggest that the combination of low-intensity rosuvastatin and ezetimibe may not be an effective strategy for preventing the elevation of HbA1c levels in patients without diabetes who require statin treatment and are concerned about the risk of diabetes during statin therapy.

## Figures and Tables

**Table 1:** Baseline patient characteristics.

		Treatment Received		
Variable	Overall, N = 68	Rosu20, n = 35	Rosu5 + Ezet10, n = 33	*p*-Value *
Age, years	62 ± 12	63 ± 12	61 ± 12	0.33
Sex, male	52 (76)	25 (71)	27 (82)	0.40
Body mass index, kg/m^2^	25.6 ± 3.1	26.1 ± 3.5	25 ± 2.5	0.11
Hypertension	33 (49)	19 (54)	14 (42)	0.33
Current smokers	18 (26)	8 (23)	10 (30)	0.49
***Indications for statin therapy***				0.87
High coronary calcium score	25 (37)	13 (37)	12 (36)	
Atherosclerosis	20 (29.5)	10 (28.9)	10 (30)	
Concurrent hs-CRP elevation	15 (22)	9 (26)	6 (18)	
Persistent LDL-C elevation	8 (11.5)	3 (8.6)	5 (15)	
***Initial laboratory findings***				
White blood cells, 10^9^/L	7.65 ± 2.84	7.48 ± 2.78	7.83 ± 2.93	0.62
Hemoglobin, g/dL	13.8 ± 1.4	13.6 ± 1.3	13.9 ± 1.4	0.34
Platelet, 10^3^/µL	224 ± 52	225 ± 57	222 ± 47	0.84
Alanine transaminase, IU/L	27 ± 18	27 ± 17	26 ± 18	0.74
Creatinine, mg/dL	1.03 ± 1.15	1.17 ± 1.59	0.87 ± 0.15	0.86
***Medication***				
Aspirin	28 (30)	14 (40)	14 (42)	0.99
ACE inhibitor	19 (28)	12 (34)	7 (21)	0.23
ARB	24 (36)	14 (40)	10 (31)	0.46
Spironolactone	9 (13)	6 (17)	3 (9.4)	0.48
***Baseline glucose metabolisms***				
HbA1c, %	5.78 ± 0.30	5.8 ± 0.31	5.83 ± 0.07	0.664
Glucose, mg/dL	124 ± 32	122 ± 24	137 ± 4	0.228
Insulin, µU/mL	9.3 ± 5.3	9.9 ± 6.3	9.9 ± 1.6	0.891
QUICKI	1.39 ± 0.29	1.41 ± 0.23	1.40 ± 0.05	0.985
***Baseline lipid profiles***				
LDL cholesterol, mg/dL	115.4 ± 37.7	114.6 ± 36.1	116.3 ± 39.9	0.515
HDL cholesterol, mg/dL	47.3 ± 11.4	45 ± 11.9	49.9 ± 10.6	0.059
Triglyceride, mg/dL	139.8 ± 70.4	153 ± 77.7	126 ± 59.6	0.088
hs-CRP, ng/dL	0.588 ± 2.81	0.247 ± 0.398	0.949 ± 4.013	0.111

Data are expressed as numbers (%) and mean ± standard deviation. * Fisher’s exact test; Wilcoxon rank-sum test; or Pearson’s chi-squared test. LDL, low-density lipoprotein; ACE, angiotensin-converting enzyme; ARB, angiotensin receptor blocker; hs-CRP, high-sensitivity C-reactive protein; HDL, high-density lipoprotein; QUICKI, quantitative insulin sensitivity check index.

**Table 2:** The means and least square means of glucose metabolism in patients receiving rosuvastatin 20 mg and a combination of rosuvastatin 5 mg and ezetimibe 10 mg at each time point.

Outcomes	Rosu20, n = 35		Rosu5 + Ezet10, n = 33			*p*
	LS Mean ± SE *	Mean ± SD	LS Mean ± SE *	Mean ± SD	LS Mean Difference (95% CI)	
***6 weeks***						
HbA1c, %	5.97 ± 0.07	5.85 ± 0.4	5.85 ± 0.07	5.74 ± 0.33	0.114 (−0.051–0.278)	0.174
Glucose, mg/dL	106 ± 4	104 ± 8	108 ± 4	105 ± 11.6	−1.967 (−11.56–7.63)	0.683
Insulin, µU/mL	13.3 ± 1.6	12.4 ± 5.7	14.2 ± 1.6	11.7 ± 9.7	−0.871 (−4.64–2.90)	0.646
QUICKI	1.56 ± 0.05	1.54 ± 0.2	1.54 ± 0.05	1.47 ± 0.27	0.026 (−0.085–0.136)	0.645
***12 weeks***						
HbA1c, %	5.96 ± 0.07	5.9 ± 0.4	5.96 ± 0.07	5.86 ± 0.31	0.018 (−0.164–0.165)	0.999
Glucose, mg/dL	105 ± 4	103 ± 8.8	106 ± 4	105 ± 13	−0.284 (−9.94–9.37)	0.953
Insulin, µU/mL	13.4 ± 1.6	14.0 ± 10.2	10.9 ± 1.6	11.8 ± 9.1	2.510 (−1.23–6.25)	0.185
QUICKI	1.55 ± 0.05	1.57 ± 0.24	1.48 ± 0.05	1.49 ± 0.25	0.049 (−0.036–0.184)	0.184

* The LS mean, SE, 95% CI, and *p*-value were calculated from multiple linear mixed-effects models that accounted for the effects of time, interaction between time and treatment, and interaction between time and baseline risk factors. LS, least square; CI, confidence interval; LDL-C, low-density lipoprotein cholesterol; HbA1c, hemoglobin A1c; QUICKI, quantitative insulin sensitivity check index; SE, standard error; SD, standard deviation.

**Table 3:** The mean and least square means of the lipid profile in patients receiving rosuvastatin 20 mg and a combination of rosuvastatin 5 mg and ezetimibe 10 mg at each time point.

Outcomes	Rosu20, n = 35		Rosu5Ezet10, n = 33			*p*
	LS Mean ± SE *	Mean ± SD	LS Mean ± SE *	Mean ± SD	LS Mean Difference, 95% CI	
***6 weeks***						
LDL cholesterol, mg/dL	61.4 ± 5.93	59.1 ± 14.9	63.6 ± 6.2	60.6 ± 30	−2.13 (−16.4–12.14)	0.766
HDL cholesterol, mg/dL	49.1 ± 2.2	47.3 ± 10.2	51.5 ± 2.3	50.2 ± 11	−2.46 (−7.79–2.863)	0.359
Triglyceride, mg/dL	125 ± 13.6	109 ± 41.1	128 ± 14.3	112 ± 68.1	−2.95 (−35.70–29.8)	0.858
***12 weeks***						
LDL cholesterol, mg/dL	64.5 ± 5.9	61.5 ± 16.4	70.7 ± 6.2	66.3 ± 29.8	−6.16 (−20.4–8.12)	0.392
HDL cholesterol, mg/dL	50.2 ± 2.2	49.2 ± 10	52.7 ± 2.3	52.3 ± 10.4	−2.42 (−7.74–2.905)	0.367
Triglyceride, mg/dL	126 ± 13.6	122 ± 43.6	128 ± 14.3	119 ± 99.5	−2.29 (−35.04–30.5)	0.890
hs-CRP, ng/dL	0.106 ± 0.419	0.167 ± 0.253	0.177 ± 0.439	0.24 ± 0.722	−0.071 (−1.08–0.937)	0.890

* The LS mean, SE, 95% CI, and *p*-value were calculated from multiple linear mixed-effects models that accounted for the effects of time, interaction between time and treatment, and interaction between time and baseline risk factors. LS, least square; HDL, high-density lipoprotein; LDL, low-density lipoprotein; hs-CRP, high-sensitivity C-reactive protein; SD, standard deviation; SE, standard error; CI, confidence level.

## Data Availability

The original protocol did not include provisions for making data and materials publicly available.
